# Impact of Particle Size on Toxicity, Tissue Distribution and Excretion Kinetics of Subchronic Intratracheal Instilled Silver Nanoparticles in Mice

**DOI:** 10.3390/toxics10050260

**Published:** 2022-05-18

**Authors:** Fernanda Rosário, Jan Creylman, Geert Verheyen, Sabine Van Miert, Conceição Santos, Peter Hoet, Helena Oliveira

**Affiliations:** 1 Department of Biology & CESAM, University of Aveiro, Campus Universitário de Santiago, 3810-193 Aveiro, Portugal; 2 RADIUS Group, Thomas More University College, Campus Kempen, Kleinhoefstraat 4, 2440 Geel, Belgium; jan.creylman@thomasmore.be (J.C.); geert.verheyen@thomasmore.be (G.V.); sabine.vanmiert@thomasmore.be (S.V.M.); 3 Department of Biology, Faculty of Sciences, University of Porto, Rua do Campo Alegre, 4169-007 Porto, Portugal; csantos@fc.up.pt; 4 Occupational and Environmental Toxicology, KU Leuven, ON1 Campus Gasthuisberg, Herestraat 49, 3000 Leuven, Belgium; peter.hoet@kuleuven.be

**Keywords:** intratracheal instillation, pulmonary exposure, mice, PBPK model, silver distribution, excretion

## Abstract

The unique physicochemical properties of silver nanoparticles (AgNPs) make them useful in a wide range of sectors, increasing their propensity for human exposure, as well as the need for thorough toxicological assessment. The biodistribution of silver, hematological parameters and GSH/GSSG levels in the lung and liver were studied in mice that were intratracheally instilled with AgNP (5 and 50 nm) and AgNO_3_ once a week for 5 weeks, followed by a recovery period of up to 28 days (dpi). Data was gathered to build a PBPK model after the entry of AgNPs into the lungs. AgNPs could be absorbed into the blood and might cross the physiological barriers and be distributed extensively in mice. Similar to AgNO_3_, AgNP5 induced longer-lasting toxicity toward blood cells and increased GSH levels in the lung. The exposure to AgNP50 increased the GSH from 1 dpi onward in the liver and silver was distributed to the organs after exposure, but its concentration decreased over time. In AgNP5 treated mice, silver levels were highest in the spleen, kidney, liver and blood, persisting for at least 28 days, suggesting accumulation. The major route for excretion seemed to be through the urine, despite a high concentration of AgNP5 also being found in feces. The modeled silver concentration was in line with the in vivo data for the heart and liver.

## 1. Introduction

The applications of silver nanomaterials are numerous and can be mainly classified under three categories: scientific, industrial and consumer products. Silver nanoparticles (AgNPs) are fast-acting fungicides [1] and effective agents against antibiotic-resistant bacteria, inhibiting biofilm formation [2,3,4]. In addition, AgNPs showed antiviral activity [5] and anti-inflammatory properties [6].

The increased manufacture and use of AgNP lead to an inevitable increase in the release of these particles into the environment through their life cycle, enabling potential exposure through various routes, including inhalation, ingestion and dermal. The exposure to dust or fumes of metallic silver and soluble silver was found to be 100 µg/m^3^ for AgNPs inhalation as an 8 h time-weighted average (TWA) concentration by the American Conference of Governmental Industrial Hygienists [7]; however, data on the human effects under realistic levels and exposure scenarios are still lacking. Studies of the toxic effects of AgNPs in animals have mainly assessed the effects of oral or systemic exposure [8,9,10,11], with a limited number reporting the effects after inhalation or intratracheal instillation. Diverse outcomes for AgNP exposure were reported, for instance, Silva et al. (2015) [12] found an increase in polymorphonuclear cells in bronchoalveolar lavage fluid at 0.5 and/or 1 mg/kg body weight (bw) after intratracheal instillation of 20 and 110 nm AgNPs stabilized with citrate or PVP. Furthermore, neutrophils, macrophages and monocytes were also found in the airway submucosa and perivascular regions on days 1 and 7 post-instillation, with 110 nm AgNPs producing lasting effects past 21 days. However, Roberts et al. (2013) [13] showed that short-term inhalation of 33 nm AgNPs (100 μg/m^3^) did not produce apparent acute toxicity in rats. Regarding the tissue distribution of AgNPs, in Sprague Dawley rats exposed to 15 nm AgNPs via inhalation, a higher concentration of Ag was found in the lungs and blood and a very low concentration was found in the liver, kidneys, spleen, brain and heart. Furthermore, the amounts of Ag in the lungs decreased rapidly with time, and by day 7, only 4% of the initial burden remained [14]. Furthermore, Sung et al. (2009) [15] found that the upper nasal deposition of 18 nm AgNPs into Sprague Dawley rats caused olfactory bulb accumulation, as well as a dose-dependent deposition of silver within the brain, blood, lungs, kidney and liver. The size and surface area of AgNPs are major determinants of the pulmonary toxicity of AgNPs and condition its clearance and biodistribution. Most studies on the biodistribution of AgNPs report the use of particles with sizes > 15 nm or >100 nm; therefore, information about the behavior of smaller particles is lacking [16]. Additionally, only the first day after the end of exposure was observed in other studies, and the long-term effects after the end of exposure were not evaluated. The elimination of AgNPs via urine and feces is addressed by a scarce number of studies. Therefore, the comparison of data from different studies is difficult, especially when different study designs were used (i.e., nanoparticle doses, animal models, administration routes and time points).

Physiologically based pharmacokinetic (PBPK) modeling could provide an insight into the relationships between an external dose and an internal organ, blood or excretion dose (IPCS 2010). Yet, for NPs, only a handful of published PBPK models are available in the literature. Lankveld et al. (2010) [9] developed a PBPK model to compare the kinetics of AgNPs 20, 80 and 110 nm in size in blood and tissues. Some of these models were extrapolated to humans and were shown to be helpful in risk assessment [16,17]. The Bachler and Hungerbühler (2013) PBPK model could successfully predict the biodistribution of silver and uncoated 15–150 nm AgNPs. The authors stated that the particle size and coating had a minor influence on the biodistribution and that, in vivo, it is more likely that AgNPs are directly stored as insoluble salt particles than dissolving into silver ions.

In the present work, we aimed at characterizing the toxicity and kinetics of distribution and excretion of AgNPs (5 and 50 nm) and ionic silver (AgNO_3_) (used as a positive control) after entry into the lungs through intratracheal instillation and fitting the data in a PBPK model. To fulfill our goals, we (I) examined hematological effects, (II) analyzed GSH and GSSG in mice lungs and liver, (III) determined the accumulation of AgNPs in target organs, (IV) evaluated the excretion of AgNPs via urine and feces, and finally, (V) analyzed the effect of AgNP size on tissue distribution at different time points.

## 2. Materials and Methods

### 2.1. AgNP and AgNO_3_ Treatments

Sterile, purified and endotoxin-free silver nanoparticles (Econix 5.0 mg/mL) with a polyvinylpyrrolidone (PVP) coating and nominal sizes of 5 nm and 50 nm (here designated as AgNP5 and AgNP50, respectively) were obtained from NanoComposix Europe (Prague, Czech Republic). The treatment solutions were prepared via dilution of AgNPs and AgNO_3_ in NaCl (0.9%) to obtain 0.075 mg for AgNPs (3 mg/kg) and 0.025 mg for AgNO_3_ (1 mg/kg). The dose of AgNO_3_ was calculated based on the silver ion mass and expressed as AgNO_3_.

### 2.2. Physicochemical Characterization of AgNPs

AgNPs were characterized previously [18]. Briefly, the morphology and size were analyzed using scanning electron microscopy (STEM) with a field emission gun Hitachi SU-70 microscope (Hitachi High-Technologies Europe GmbH, Krefeld, Germany) operating at 15 kV. Approximately 10 μL of each stock solution was added to a carbon sheet and left to dry in atmospheric conditions. After the samples were dry, the STEM images were taken and analyzed with KLONK Image Measurement software (https://www.imagemeasurement.com/en/, accessed on 27 May 2020). The hydrodynamic diameter, polydispersity index and zeta potential of AgNPs were measured after preparation in saline (NaCl 0.9%) and water using Dynamic light scattering (DLS) with a Malvern Zetasizer Nano ZS (Malvern I, UK). Additionally, the absorbance spectra of the AgNPs suspensions were obtained over a range of wavelengths in the visible light range (280 to 800 nm) using a Thermo Scientific Evolution 220 spectrophotometer at 100 scans/min with a bandwidth of 2 nm and an integration time of 0.3 s.

### 2.3. Mice

Male BALB/c OlaHsd mice (6 weeks old—20 g) were obtained from Harlan (Horst, The Netherlands). The mice were housed in a conventional animal house with 12 h dark/light cycles. They were housed in filtertop cages and received lightly acidified water and pelleted food (Trouw Nutrition, Ghent, Belgium) ad libitum. Individual animal weights were recorded every week pre-dosing and at the time of death or euthanasia. All experimental procedures were approved by the local Ethical Committee for Animal Experiments (License KU Leuven 054/2015).

### 2.4. Study Groups and Dosing Regimen

**Figure 1:**
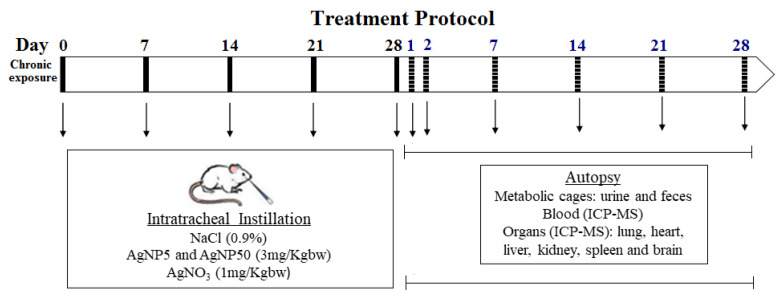
Schematic overview of the experimental protocol. Black lines mean exposure time and dashed lines mean recovery time.

The experimental protocol was divided into two main time windows: 28 days of exposure and 28 days of recovery with a total of 56 days. Ninety-six mice were divided into three experimental groups (AgNP5/AgNP50/AgNO_3_) and one control group (0.9% NaCl) per each recovery time point (1, 2, 7, 14, 21 and 28 days post instillation), with four mice in each. On days 0, 1, 7, 14, 21 and 28 of the exposure time, mice were intratracheally instilled with treatment solutions (150 µL of air + 50 µL of NaCl/AgNP5/AgNP50/AgNO_3_ solutions), once a week for 5 weeks. The solution was administered using 24-gauge catheters under intraperitoneal anesthesia that was a mixture of xylazine + ketalar in 0.9% NaCl (150 µL/25 g of body weight). The recovery period began at the end of the last exposure. The mice were checked for normal behavior and placed back in the cages. On days 1, 2, 7, 14, 21 and 28 post the last instillation (dpi), the mice were weighed and sacrificed using an intraperitoneal injection of 200 µL of Nembutal. A schematic overview of the experimental protocol is shown in Figure 1.

### 2.5. Blood Collection and Analysis

After euthanasia, blood was collected from the retro-orbital plexus. Then, 500 µL of blood was collected into a microtube with 50 µL of citrate (3.8%) and diluted in 200 µL of sterile saline (0.9% NaCl). Total and differential blood cell counts (white blood cells, neutrophils, monocytes, lymphocytes, eosinophils, basophils, red blood cells, platelets), hemoglobin concentration and mean corpuscular volume were determined on a Cell-Dyn 3500R counter (Abbott Laboratories, Taguig, Philippines).

### 2.6. Assessment of GSH and GSSG Levels

The levels of oxidized (GSSG) and reduced (GSH) glutathione were determined according to [19] with some modifications. Lung and liver samples were collected, snap-frozen and stored at −80 °C until analysis. Lung and liver samples were homogenized on ice with cold 40 mM N-ethylmaleimide (20 mL/g tissue to prevent rapid oxidation of GSH) and then centrifuged at 14,000× *g* for 15 min at 4 °C. The supernatant was transferred to a new tube and 5% metaphosphoric acid was added (1/5th of the supernatant volume, final concentration was 1% metaphosphoric acid, for removing the proteins), mixed and again centrifuged at 14,000× *g* for 15 min at 4 °C. The supernatants were stored at −80 °C until their analysis. Total protein levels were determined using the Bio-Rad Protein assay according to the Bradford method. The ratio of GSSG to total glutathione was then calculated (GSSG:GSH) (BioRad Laboratories GmbH, München, Germany, using bovine serum albumin as the standard).

### 2.7. Silver Quantification Using ICP-MS

At 1, 7 and 28 dpi, samples from the following organs were collected: lung, blood, hair, spleen, kidney, liver, brain and bone marrow for silver quantification using inductively coupled plasma mass spectrometry (ICP-MS). For the same days, 4 mice from each treatment (AgNP5/AgNP50/AgNO_3_ and control (0.9% NaCl)) were placed in metabolic cages for 24 h to collect urine and feces. Samples for ICP-MS analysis were digested in Aqua Regia. Briefly, 1.5 mL HCl and 0.5 mL HNO_3_ were added to approximately 30 mg tissue or 100 µL urine in glass tubes and left for 24 h. After 24 h, samples were heated at 140 °C for 90 min. Finally, samples were diluted in Milli-Q water to 10 mL and Ag concentration was measured using ICP-MS (Agilent 7700x ICP-MS). Elements were measured as 107 Ag using 103 Rh as the internal standard. The detection limit was 0.015 µg Ag/L.

### 2.8. Modelling PBP Model

The structure of the PBPK model was based on the available knowledge of the disposition of ionic silver and AgNP within rats and humans, as described by Bachler et al. (2013) [16]. It was assumed that the mechanisms of absorption, distribution, metabolism and excretion (ADME) follow first-order kinetics, which simplified the model; this is also common for many other PBPK models. Physiological parameter values of mice were extracted from [20,21]. The absorption of nanoparticles through instillation was simulated based on a simplified International Commission on Radiological Protection (ICRP) Human Respiratory Tract Model, as shown in supplementary materials from Bachler et al. (2013) [16]. It was assumed that 100% of the instilled AgNPs were deposited in the initial compartment of the lungs. Through this compartment, the nanoparticles were transported into the blood and then distributed to all tissues. However, a fraction of the instilled dose was stored in a “bound state”, and other fractions directly moved to the gastro-intestinal tract or were exhaled instantaneously. Except for the “remaining tissues” and blood compartments, each of the compartments were divided into two subcompartments. A first subcompartment represented the AgNP particles, which could freely exchange between blood and tissue. The other subcompartment was assumed to store the instilled particles in the tissue. The uptake of AgNPs in the mononuclear phagocyte system (MPS) of the lungs, liver and spleen was not included in this model because the blood silver concentration did not exceed 180 ng/g [16]. The bone marrow was assumed to be a sink for all AgNPs that were neither distributed to organs in the model nor excreted. For this reason, particle release from the bone marrow to the blood was not implemented in the model. Due to the lack of mouse data, uptake constants of silver nanoparticles from blood to various tissues were taken from the rat model. These constants describe the membrane transport of AgNPs and were assumed to be size-independent. The release rate to the blood was assumed to be the same for all tissues. The distribution of nanoparticles in the bloodstream was also assumed to be size-independent. Particle dissolution into smaller particles or ionic silver was neglected to simplify the model. The biliary and urinary excreted silver nanoparticles were directly removed from the blood compartment. RStudio© (Version 0.98.1103, Boston, MA, USA) [22] was used to set up the model. The deSolve package was used for solving differential equations [23]. To simulate the five weekly instillations, the events parameter of the ode function was addressed.

### 2.9. Statistical Analysis

The results are reported as mean ± standard deviation (SD). Statistical analysis was performed in the SigmaPlot version 11 software (Systat Software Inc., Palo Alto, CA, USA). Data were tested for normality and homogeneity of variances using Shapiro–Wilk and Levene’s tests, respectively. For each timepoint, differences between the tested concentrations and control were estimated using one-way and two-way ANOVA analysis of variance (*p* < 0.05), followed by the Holm–Sidak test or Dunn’s test for the parametric and non-parametric data, respectively. The differences were considered statistically significant for *p* < 0.05.

## 3. Results

### 3.1. AgNPs Characterization

The AgNPs’ morphology, size, dispersion and charge were characterized using STEM, DLS and spectrophotometry, as described previously [18]. Briefly, the physicochemical data showed that the AgNPs’ diameters determined using STEM were 5.44 ± 1.05 for AgNP5 and 52.04 ± 6.05 nm for AgNP50. The hydrodynamic diameters in saline solution (Dh) were 39.29 ± 0.89 for AgNP5 and 304 ± 30.26 nm for AgNP50, with polydispersity indexes (PdI) of 0.493 ± 0.058 and 0.481 ± 0.070, respectively, for AgNP5 and AgNP50. The zeta potentials (ζ) of AgNP5 and AgNP50 in saline solution were −13.9 ± 0.95 and 1.42 ± 0.94 mV, respectively.

### 3.2. Mice Clinical Health Observations

Overall, the repeated intratracheal instillations of AgNPs and AgNO_3_ were well-tolerated by the animals and normal feeding behavior was observed. The animals did not exhibit any signs of illness during the instillation or the recovery period. Two out of forty-eight animals died outside of the planned sacrifices: one was euthanized for unrelated causes (abnormal abscesses found in the heart) and the other one died because of an intratracheal-related injury. No unpredicted deaths were observed due to the tested substances. An interesting remark is that in the last week of treatment, the mice treated with AgNP50, and especially AgNO_3_, showed altered sexual behavior toward the same sex. Alterations of the accessory olfactory bulb or stress/anxiety could be responsible for the same-sex attraction.

### 3.3. Body and Organ Weights

**Figure 2:**
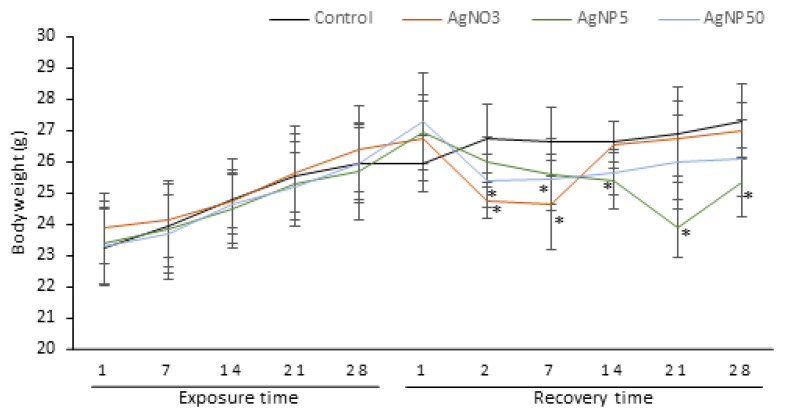
Mice body weight changes during a 28-day exposure via intratracheal instillation of saline (control), AgNO_3_, AgNP5 and AgNP50 and a 28-day recovery time. * means significant differences vs. last day of exposure (one-way ANOVA; Holm–Sidak *p* ≤ 0.05) (*n* = 4).

**Figure 3:**
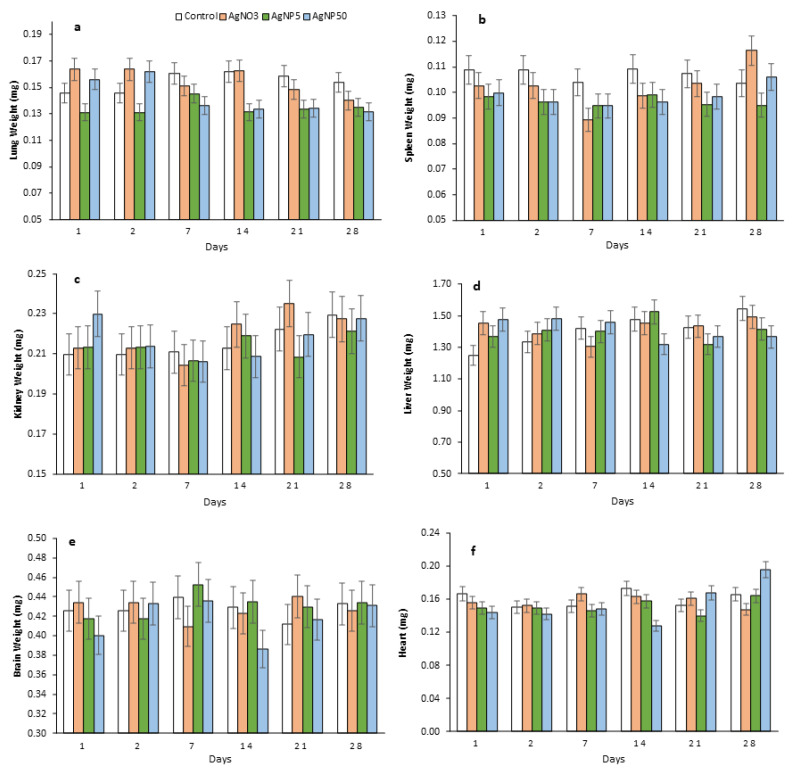
Mice relative organ weights over a 28-day recovery time after repeated intratracheal instillations of saline (control), AgNO_3_, AgNP5 and AgNP50. (**a**) WBC; (**b**) lymphocytes; (**c**) neutrophils; (**d**) monocytes; (**e**) eosinophils; (**f**) basophils.

No significant body weight (bw) change was observed during the exposure time (Figure 2). However, after the cessation of exposure, the mice’s bw fluttered and decreased, except for in the control group. The AgNO_3_ treatment significantly decreased the mice’s bw at 2 and 7 dpi, but after that, the mice seemed to recover well and their bw was in line with the control at 14 dpi. AgNP5 showed a significant bw decrease from 7 to 28 dpi. The AgNP50 treatment significantly decreased the mice’s bw at 2 dpi, recovering slowly after that time (Figure 2). No significant organ weight changes were observed in either control or treated mice after 28 dpi—or any other time-point considered—due to Ag and AgNPs exposure (Figure 3).

### 3.4. Blood Parameters

**Figure 4:**
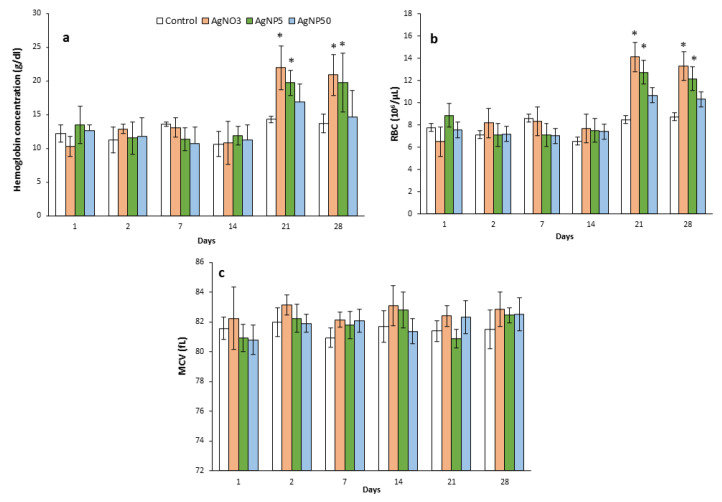
Differential mice blood cell counts for hemoglobin concentration (**a**), RBC (**b**) and MCV (**c**) over a 28-day recovery time after repeated intratracheal instillations of saline (control), AgNO_3_, AgNP5 and AgNP50. * means significant differences between the control and treatments (one-way ANOVA; Dunn’s test *p* < 0.05) (*n* = 4).

**Figure 5:**
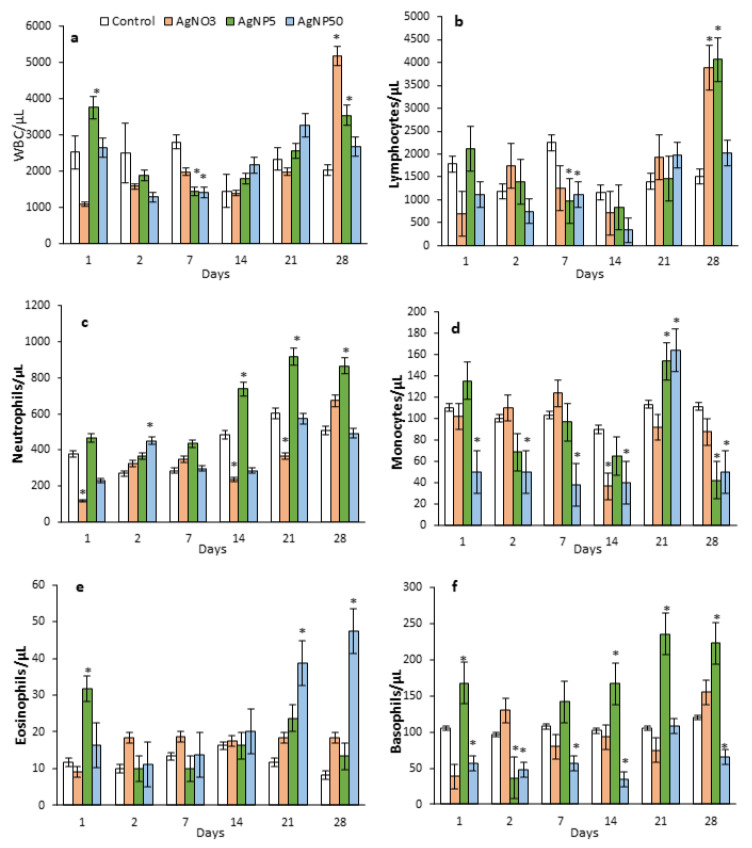
Mice hematology values during a 28-day recovery time after repeated intratracheal instillations of saline (control), AgNO_3_, AgNP5 and AgNP50. (**a**) WBC; (**b**) lymphocytes; (**c**) neutrophils; (**d**) monocytes; (**e**) eosinophils; (**f**) basophils. * means significant differences between the control and treatments (one-way ANOVA; Dunn’s test *p* < 0.05) (*n* = 4).

Hematology results are depicted in Figure 4 and Figure 5. At 21 and 28 dpi for AgNO_3_ and AgNP5 exposures, significant increases in hemoglobin (HGB) concentration and the number of red blood cells (RBC) were observed. No significant statistical differences were observed in the mean corpuscular volume (MCV) for any treatment. AgNP50 decreased the number of basophils and monocytes at all timepoints, except for 21 dpi (Figure 5d,f). Contrarily, a significant increase in the number of basophils was observed for AgNP5 exposure at 1, 14, 21 and 28 dpi (Figure 5f). Total white blood cells (WBC) were only affected at 7 and 28 dpi by AgNP5 and AgNO_3_ exposures, which decreased and increased, respectively (Figure 5a). Neutrophils were significantly decreased by AgNO_3_ at 1, 14 and 21 dpi, while AgNP5 exposure increased the number of neutrophils from 14 dpi until the end of recovery (Figure 5c). The AgNO_3_- and AgNP5-exposed groups showed a significant increase in the total number of lymphocytes at 28 dpi, while at 7 dpi, the number of lymphocytes was decreased due to AgNP5 and AgNP50 exposure. The minimum number of lymphocytes was obtained at 14 dpi due to AgNP50 exposure (Figure 5b). At 1 dpi, the number of eosinophils was increased by AgNP5 and recovered from there on, while AgNP50 increased the number of eosinophils from 21 dpi, reaching a peak at 28 dpi (Figure 5e). 

### 3.5. GSH and GSSG Levels

**Figure 6:**
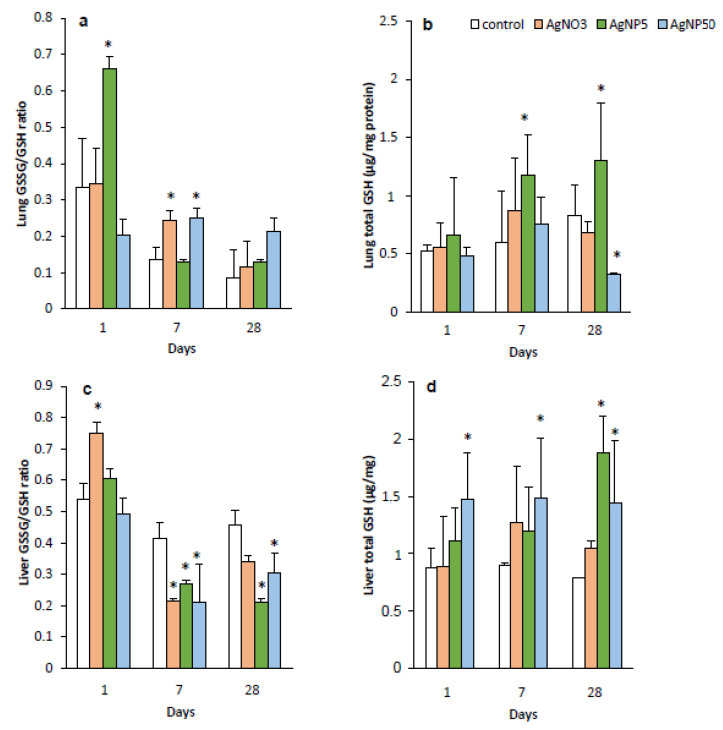
GSH and GSSG levels (µg/mg protein) and GSSG:GSH ratio analysis during a 28-day recovery time after repeated intratracheal instillations of saline (control), AgNO_3_, AgNP5 and AgNP50. (**a**) Lung GSSG:GSH ratio; (**b**) lung total GSH; (**c**) liver GSSG:GSH ratio; (**d**) liver total GSH. * means significant differences between the control and treatments (one-way ANOVA; Dunn’s test *p* < 0.05) (*n* = 4).

The results of the GSH and GSSG:GSH ratio quantification in the lung and liver tissues are presented in Figure 6. The GSSG:GSH ratio was significantly increased at 1 dpi for AgNP5 (Figure 6a), while for AgNP50 and AgNO_3_, the GSSG:GSH ratio was increased at 7 dpi at the expense of the GSH content (Figure 6b). Additionally, in the lung, the GSH content was increased for AgNP5 exposure at 7 and 28 dpi, and for AgNP50 treatment, the GSH content decreased at the last dpi. Finally, AgNO_3_ did not alter the redox state of the lung.

Regarding the liver, the GSSG:GSH ratio was significantly increased for AgNO_3_ after 1 dpi, followed by a decrease at 7 dpi and was almost similar to the control at 28 dpi. 

The exposure to both types of AgNPs decreased the GSSG:GSH ratio levels at 7 and 28 dpi (Figure 6c). The levels of GSH in the liver presented an increasing behavior from day 1 for AgNP50, while AgNP5 increased the GSH levels at 28 dpi (Figure 6d).

### 3.6. Biodistribution, Accumulation and Elimination of AgNPs

**Figure 7:**
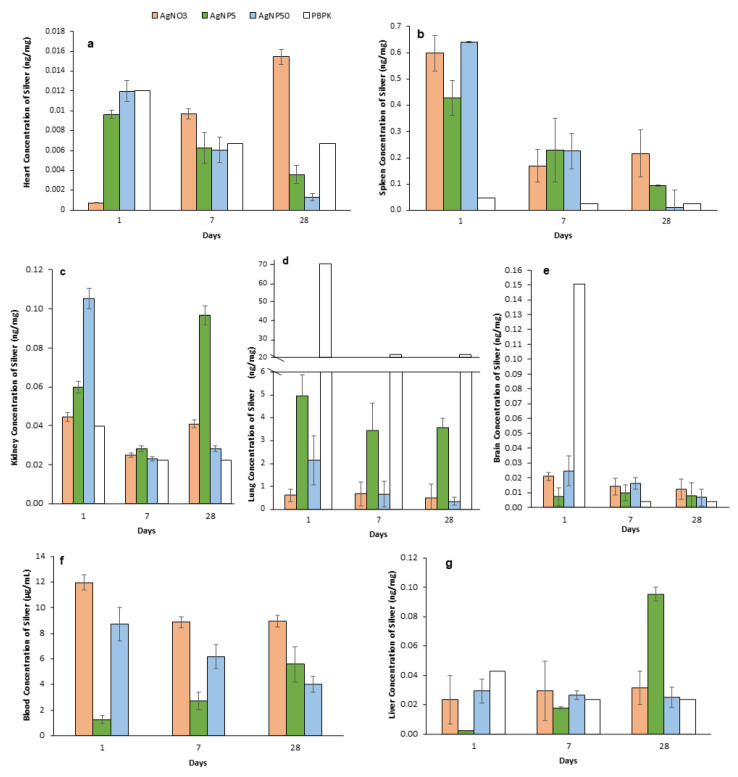
Silver concentrations (ng/mg tissue fresh weight) during a 28-day recovery time after repeated intratracheal instillations of saline (control), AgNO_3_, AgNP5 and AgNP50. (**a**) Heart; (**b**) spleen; (**c**) kidney; (**d**) lung; (**e**) brain; (**f**) blood; (**g**) liver. Silver PBPK data are represented as the lighter bars (*n* = 4).

Total Ag contents were determined in the selected organs (lung, brain, heart, spleen, kidney, liver and bone marrow), as well as in urine, feces and blood using ICP-MS. Unfortunately, the Ag concentrations in bone marrow and hair were below the detection limit of ICP-MS due to the small amount of sample collected. In control samples, Ag levels were also below the detection limits and, therefore, data are not presented for this group. Figure 7 shows the mean silver levels in the organs after 1, 7 and 28 dpi. The results showed that at 1 dpi, the highest levels of silver were detected in the lungs (Figure 7d), urine and blood (Figure 7f), followed by the spleen and kidney, while the least accumulation was found in the heart and liver. Concerning AgNPs, they were translocated from the lungs to the other organs analyzed, as silver was detected in all organs evaluated, regardless of particle size.

In the lungs, at 1 dpi, the highest levels of silver were observed for the AgNP5 treatment. For this treatment, the concentration of silver at 1 dpi in the lung was 4.9 ng/mg tissue and was still high until 28 dpi. The concentration of silver for AgNP50 was 2.2 ng/mg tissue and deceased after that until almost total clearance at 28 dpi (0.04 ng/mg tissue) (Figure 7d). The same pattern of clearance, from 1 to 28 dpi, was observed in the brain, spleen, kidney, liver and blood for AgNP50 (Figure 7). The smaller particles (AgNP5) showed a somewhat opposite silver level distribution compared with AgNP50. For instance, for AgNP5 treated mice, the silver concentrations in kidney, liver and blood were highest at 28 dpi, while in the heart and brain, silver decreased from 1 to 28 dpi (Figure 7a,e).

For the AgNO_3_ treatment, the highest concentrations were observed in the lung, blood, kidney and heart (Figure 7). Except for the heart, the concentration of silver in all organs of mice treated with AgNO_3_ decreased until 28 dpi, but no total clearance was observed. In the post-instillation time, the silver concentration decreased faster from 1 dpi to 28 dpi in almost all organs for treatments with AgNP50 and AgNO_3_; however, at the end of the recovery time (28 dpi), a small concentration of silver remained (Figure 7). In contrast, for the AgNP5 treatment, the elimination seemed to occur at a slower rate, where it was possible to observe a high concentration of silver in several organs at 28 dpi (Figure 7b,c,g). Animals exposed to AgNO_3_ and AgNP50 showed similar concentrations of silver in the liver at 7 and 28 dpi (Figure 7g).

### 3.7. Excretion of AgNPs

**Figure 8:**
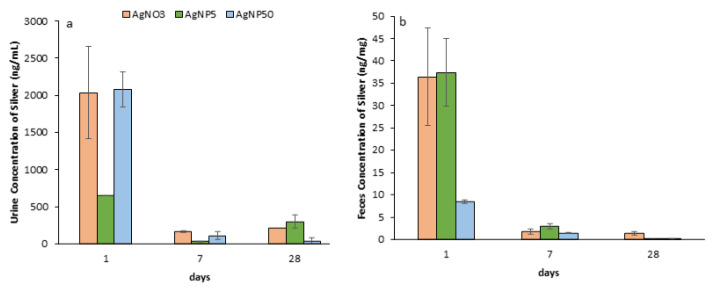
Excreted silver concentrations (ng/mL or ng/mg) during a 28-day recovery time after repeated intratracheal instillations of saline (control), AgNO_3_, AgNP5 and AgNP50. (**a**) Urine; (**b**) feces (*n* = 4).

The kinetics of silver excretion in urine and feces was determined by measuring the silver concentration in urine and feces samples collected from animals kept in metabolic cages for 24 h at 1, 7 and 28 dpi. Figure 8a,b show that a major part of the silver was excreted via the urinary tract and the highest silver levels in the urine were obtained for mice exposed to AgNP50 and AgNO_3_. The highest concentrations of silver in feces were obtained for AgNP5 and AgNO_3_ treatments at 1 dpi (Figure 8b). The nanoparticle elimination rate in feces decreased steadily and reached the minimal value at 28 dpi.

### 3.8. PBPK Model

Figure 7 shows the results of the PBPK model compared with the experimental results of both 5 and 50 nm nanoparticles. The ADME model was created to validate and help to predict the amount of silver in each compartment (Figure 7 and Figure 8). Modeling the ADME of AgNPs in seven specific tissues of mice was found to be successful for the heart and liver compartments. For these compartments, the modeled silver concentrations seemed to be in line with the experimental data. However, the modeled silver levels in the lung compartment seemed to be overestimated, as AgNPs seemed to be more quickly removed from the lungs. The absorption of AgNPs through instillation may need a different approach than the simplified ICRP Human Respiratory Tract Model. Experimental data of silver levels in the brain showed that the concentration did not exceed 0.05 ng/mg organ at 1 dpi. However, the modeled silver level was close to 0.15 ng/mg organ. This could show that the actual uptake of silver in the brain was much lower or the release rate to the blood was much higher than simulated. For the spleen, the opposite was true, where the in vivo data showed a much higher uptake or slower release rate, or even a higher storage capacity than the simulated spleen compartment.

## 4. Discussion

In the present work, the toxicity, distribution and excretion of two differently sized AgNPs (and ionic silver) were assessed in a mouse model after repeated intratracheal instillation exposures. Exposure via intratracheal instillation allowed for the study of lung toxicity of substances with the advantage of not requiring the specialized facilities that are required for inhalation studies, reducing the inherent costs of the studies. Additionally, the toxicity induction and clearance of AgNPs can be similar to an inhalation study, if the dosage is not excessive [24].

Given that the workplace exposure limit for silver dust and fumes is 100 µg/m^3^ and the average adult working male breathes 16.8 m^3^ of air per day, this would yield a daily exposure of 1.68 mg of silver/day [25,26]. The dose tested in this study was 3 mg/kg for AgNPs; therefore, for a 25 g mouse, the total exposure was 0.375 mg per mouse, which is well within the range of potential acceptable workplace exposure. Since the burden of particulate silver is higher than the one caused by AgNPs, we used a lower dose of AgNO_3_ administered to mice (1 mg/kg).

Exposure to AgNPs significantly decreased the bw of mice during the recovery period, mostly for animals exposed to AgNP5. A decrease in bw upon AgNPs exposure to smaller AgNP was also described by Shahare and Yashpal (2013) [27] and by Recordati et al. (2016) [28]. These authors hypothesized that the weight decrease was related to a loss of microvilli and reduced absorptive capacity of intestinal epithelium induced by AgNPs [27]. Considering the high amount of silver excreted by feces at 1 dpi, a possible alteration in intestinal function could have occurred upon AgNP5/AgNO_3_ exposure. We could speculate that the exposure to AgNP5 accumulated in mice had more sustained effects and higher toxicity than that of AgN50 on mice growth.

Subchronic instillations of AgNPs affected the mice hematology, clearly demonstrating a particle size dependence on the induction of toxic effects on mice blood cells. Most studies on AgNPs toxicity toward blood cells reported an increase in membrane damage to RBC with subsequent hemolysis [29,30]. In contrast, our study showed an increase in RBC and HGB in mice exposed to AgNO_3_ and AgNP5, indicating a need to compensate for any condition that resulted in low oxygen levels via pulmonary disruption. In our study, a marked size-dependent reaction of the immune system was observed as a response to exposure to AgNP5, AgNP50 and ionic silver. Exposure to AgNP5 and AgNO_3_ increased the number of neutrophils, basophils and lymphocytes in exposed mice from 14 to 28 dpi, indicating a long-lasting inflammatory response. Our results are in agreement with other reports, which investigated lung inflammation in the BALF and reported neutrophilic influx as an inflammation marker. This is especially observed for smaller sizes of AgNPs, which are easily engulfed by macrophages, activating pro-inflammatory responses [31]. Although it is still controversial, several studies reported that smaller particles induced higher production of IFN-α, TNF-α and GM-CSF cytokines than larger particles in vitro, as well as stronger inflammatory responses [32,33,34].

In our experiments, AgNP5 showed similar effects to ionic silver exposure, suggesting that the effects induced from AgNP5, apart from its small size, could be derived from silver release. The exposure to AgNP50 induced toxic effects in monocytes, with a highly marked decrease in the number of monocytes through the entire recovery time, which was perhaps caused by a monocyte uptake of the larger particles (large aggregates > 300 nm were observed in the DLS data), blocking its differentiation or leading to frustrated phagocytosis, followed by failed clearance and the recruitment of eosinophils [35,36]. Contrarily to neutrophils, which are involved in the phagocytic response to small particles, eosinophils release inflammatory molecules and cytotoxic cationic proteins to target larger particles. Similarly, the detection of eosinophilia upon exposure to AgNP50 was in agreement with these findings.

GSH is used to protect mammalian cells against oxidative damage and the reduced and oxidized forms of glutathione (GSH and GSSG, respectively) act in concert to regulate and maintain the cellular redox status. Under normal conditions, more than 95% of the glutathione (GSH) in a cell is reduced; however, under oxidative stress conditions, GSH is oxidized to GSSG, and thus, the GSSG:GSH ratio is altered [37]. The depletion of GSH will lower the reducing capacity of the cell and can therefore induce oxidative stress. Oxidative stress induced by NPs could consequently cause damage to cell membranes and tissues while enhancing inflammation [38]. Regarding the lung data, the highest effects were observed for AgNP5 > AgNP50 > AgNO_3_. Although the effects were not always correlated with the levels of silver present in the organ, the increase in the GSH levels in the lung and liver after AgNP5 exposure could reflect the accumulation of silver content observed in the lung (7 and 28 dpi) and liver (28 dpi). Furthermore, the GSSG:GSH ratio content in the lungs of mice exposed to AgNP5 first increased and then decreased with time, indicating that the content of oxygen free radicals increased in the lung and GSH increased to scavenge free radicals. Similar results were found by Gan et al. (2020) [39]. Regarding the liver, higher concentrations of GSH were found, but since the liver is the main site for GSH synthesis, these results were expected. Increased GSH levels from 1 to 28 dpi, followed by a decrease in the GSSG:GSH ratio, were found for AgNP50, which clearly indicated oxidation of the GSH as a stress response right after the end of the exposure time.

Concerning the NP tissue distribution and accumulation, some studies reported a distinct size-dependent distribution [28], while others claimed that the silver distribution is irrespective of the size [40]. Following intravenous administration (IV), NPs are rapidly and widely redistributed to various systems, but the majority would be taken up by the liver via the first-pass effects and then redistributed from the liver to the other organs [41]. Several studies suggested that after an IV, NPs are distributed to the colon, lungs, bone marrow, liver, spleen and lymphatic system, followed by a rapid clearance by the liver and splenic macrophages [42]. Such a distribution is followed by rapid clearance from the systemic circulation, predominantly by the action of the liver and splenic macrophages [43]. For instance, inhalation and intratracheal instillation studies showed that inhaled or intratracheally instilled ultrafine particles of iron oxide or titanium dioxide were found mainly in alveolar macrophages. Then, NPs effectively penetrated the circulatory system and were deposited extensively in the heart, liver, spleen, lung, kidney, brain, stomach, small intestine and bone marrow, with the highest concentrations detected in the lung, liver and spleen [44]. For instance, Ferdous et al. (2021) [45] found that 10 nm AgNPs were distributed mainly in the spleen, liver and lung, as well as a little in the kidney and brain, after 7 days of a single exposure via intratracheal instillation. In our work, a higher amount of silver was observed in the blood, followed by the lung, spleen, kidney, liver, brain and heart. Hence, the amount of silver in the blood could have been overestimated since as much blood as possible was collected during the autopsy to maximize the removal of residual blood from the organs, possibly increasing the content of silver in the blood [9]. Our work showed that animals exposed to AgNP50 presented silver translocation to the bloodstream and consequent distribution to the target organs similar to mice exposed to AgNO_3_, with an almost total clearance at 28 dpi. Fehaid et al. (2016) [46] recovered 1% of the silver in mice exposed to AgNO_3_ at 28 days after one single instillation, suggesting that the silver ions are easily absorbed into the circulation and distributed to different tissues more than the nanoparticles. Moreover, Rosário et al. (2020) [18] demonstrated that AgNP50 was more efficiently cleared from the lungs than AgNP5 after acute exposure. Taken altogether, it is possible to say that larger particles, as well as AgNO_3_, have low persistence in the tissues, although it is difficult to predict whether the silver distributed from mice exposed to AgNP50 was in the ionic form or as NPs.

AgNP5 showed an opposite profile of distribution after intratracheal instillation with high levels of silver at 28 dpi in the lungs, spleen, kidney and liver. These organs were the main accumulating sites. The exceptions were the heart and brain, which showed a decrease in silver concentration over time. The delayed distribution of AgNP5 from 1 to 7 dpi could have also occurred. Previous studies showed that the smaller-sized nanoparticles could enter the interstitial space through some epithelial barriers and be entrapped by the macrophages with a slow release of silver and accumulation for a long time [46].

Nevertheless, to our knowledge, this is the first study on subchronic silver biodistribution of 5 nm AgNPs; therefore, the ability to compare our results with those in the literature is limited. Comparing the inhalation silver exposure studies is also problematic due to differences in exposure levels, particle size and surface coatings, as well as the mechanism of aerosol generation.

To our knowledge, the excretion of AgNPs in inhalation or intratracheal instillation studies was confined to one study in mice and one in humans exposed to silver [47,48]. Chuang et al. (2013) [47] reported trivial amounts of 33 nm AgNPs excreted in urine and feces of mice up until 7 dpi after inhalation, while DiVincenzo et al. (1985) [48] found that silver was eliminated predominantly via feces. The kidney is capable of rapidly removing NPs with minimal involvement of intracellular catabolism, reducing the possibility of retention and cytotoxicity compared with the hepatobiliary system. The filtration size threshold for nanoparticles is ideally 4.5–5 nm in diameter [49]. Particles that do not undergo renal clearance are ultimately excreted through the hepatobiliary system, although particles cleared by the liver are catabolized first, leading to this excretion pathway becoming more complex. The accepted scale for the capture and clearance of particles from the liver is 10–20 nm [50]. In the present study, the excretion of AgNPs through urine or feces seems to be dependent on the AgNP size. The highest silver excretion through urine was observed at 1 dpi for AgNP50 and AgNO_3_, which was consistent with a higher concentration of silver observed in the kidney of mice treated with AgNP50 at 1 dpi. A faster particle dissolution into ionic silver for AgNP50 could be the reason for the excretion via urine [9]. Finally, silver excretion via feces was higher for mice exposed to AgNP5 and AgNO_3_. In the case of exposure to AgNP50, the silver levels excreted by feces were lower and silver levels in the liver were maintained over time. This shows a high accumulation of silver in the liver due to the difficulty of excretion of larger particles by the bile duct. In animals exposed to AgNP50, the levels of silver excreted through feces were the lowest from all treatments, while the highest excretion of silver through feces was verified for AgNP5 and AgNO_3_ at 1 dpi.

To the best of our knowledge, no PBPK models have been published for the distribution of inhaled (5–50 nm) AgNPs in mice. Therefore, we implemented a PBPK model developed by Bachler et al. (2013) [16] that was based on the available knowledge on the disposition of nanosilver within rats, which was validated using published data. In the translation of the Bachler et al. model, the physiological parameters of mice were implemented as much as possible. As described in the methods section, several assumptions underlie the PBPK model that may impact the fit of the model to the data. The modeled silver levels in heart and liver compartments for AgNP from 5 to 50 nm were in line with the in vivo data, while in the lung compartment, the modeled silver level was much higher than the experimental values. These differences may be explained by the different administration procedures that were followed. Our assumption in the model was that all instilled AgNPs were absorbed in the “initial state” compartment defined in the lungs. Our experimental data indicated that the modeled release out of the lungs into the blood compartment may have been too little and should be higher in mice. Alternatively, a future version of the PBPK model may also implement an exhaled fraction, a fraction of the instilled AgNPs that may be translocated due to mucociliary clearance and a fraction that may be sequestered and degraded by macrophages [51]. The blood–brain barrier (BBB) plays an important role in the transport of molecules between the blood and brain compartments; however, the BBB was not considered in our version of the model, which could explain the overestimation of silver levels in the brain compartment at 1 dpi. The spleen compartment also plays an important role in the MPS, but the latter was not incorporated into the PBPK model, which could explain the lower modeled silver levels in the spleen. The modeled uptake, release and storage rates of AgNPs used data from rats, as described by Bachler et al. (2013) [16]. Using specific rates for mice could enhance the prediction ability of the model. These rates can be estimated through reverse engineering using system identification and parameter estimation [52]. However, this implies a more extended in vivo data set on an hourly basis, especially within the first day post-exposure. Finally, the decomposition of AgNP into both smaller-sized nanoparticles and ionic silver could also explain the lower estimated levels of silver nanoparticles in the various compartments. Similar to Lankveld et al. (2010) [9], the model did not indicate clear relationships between the AgNP size and its corresponding kinetic characteristics. Toxicokinetic data of AgNPs smaller than 15 nm is highly necessary since there is no information for this size range [16]. Our observations suggest different kinetics between 5 nm and 50 nm AgNPs (e.g., in the kidney and blood). Although, in contrast to larger particles, the direct passage of very small NPs (<15 nm) through the pores of the organ blood capillaries is expected, the clearance through the kidneys is also expected to be more important for particles less than 8 nm in size and hepatobiliary clearance is the main mechanism of excretion for larger nanoparticles [52]. However, our experimental observations gave a more complex picture because kidney silver levels of AgNP50 were higher than AgNP5 at 1 dpi but lower at 28 dpi. Therefore, future versions of the PBPK model must implement the mechanisms that may underlie differences in kinetics and tissue distribution that can be attributed to the NP size. Other physicochemical characteristics besides size may also affect nanoparticle kinetics, such as the surface charge, surface coating, number of particles, protein absorption and the tendency of aggregation/agglomeration in plasma. This implies the use of interactive, and thus, much more complex modeling algorithms and experimental data to allow for the generation of hypotheses that may explain specific mechanisms that underlie the kinetics, as well as experimental data that will allow for a proper validation of models.

## 5. Conclusions

Overall, we assessed the toxicity and distribution of two distinct sizes of AgNPs at different timepoints (1, 2, 7, 14, 21 and 28 days) in an in vivo model.

We demonstrated that the effects of these nanoparticles were size-dependent, and thus, might be associated with a different health risk. Ion dissolution seemed to play an important role when assessing the kinetics and effects of AgNPs, although the outcomes of AgNP exposure may not only be attributed to the release of Ag^+^ ions but also to the nanoparticulate form. Smaller particles seemed to induce long-lasting inflammatory effects with a high influx of neutrophils until the last day of recovery (28 days). Since these effects were similar to that of the AgNO_3_ exposure, the particulate silver seemed to be responsible. AgNP5 was distributed extensively in mice and accumulated in specific organs, such as the liver, kidney, spleen and lung, persisting for at least 28 days. Larger particles may have been differently detected by the immune system, which involved the recruitment of eosinophils (larger phagocytic cells). Furthermore, AgNP50 presented a similar profile of distribution to AgNO_3_, with a high clearance at 28 dpi. At last, the excretion of AgNPs was once again determined by the size. The major route for excretion seemed to be through the urine, despite a high concentration of AgNP5 also being found in feces.

Moreover, after crossing the physiological barriers, AgNPs can translocate into the systemic circulation, causing oxidative stress to secondary organs. The increase in the GSSG:GSH ratio and GSH content could have been a response to the cell antioxidant needs in the lung, while the cellular redox status in the liver could be related to a stress response and to biliary excretion of silver as a Ag-GSH complex. Modeling the ADME of AgNPs in seven specific tissues of mice was shown to be successful for the heart and liver. The following experimental studies would help to further increase the reliability of PBPK models: (1) determination of particle dissolution into smaller particles or ionic silver; (2) investigations into the deposition of AgNPs by the bone marrow; (3) storage kinetics of ionic silver and AgNP, including the influence of macrophages; and (4) discrimination between models for the nanoparticle lower and upper size limits. The data from this research provided information on the toxicity and biodistribution of AgNPs following lung administration in mice and might shed light on the future application of AgNPs in daily life.

## Data Availability

Not applicable.
